# The Relationship Between Employees’ Daily Customer Injustice and Customer-Directed Sabotage: Cross-Level Moderation Effects of Emotional Stability and Attentiveness

**DOI:** 10.3389/fpsyg.2022.819396

**Published:** 2022-03-07

**Authors:** Young Ho Song, Jungkyu Park

**Affiliations:** ^1^Odette School of Business, University of Windsor, Windsor, ON, Canada; ^2^Department of Psychology, Kyungpook National University, Daegu, South Korea

**Keywords:** daily customer injustice, daily customer-directed sabotage, emotional stability, attentiveness, cross-level moderation effects

## Abstract

Customer injustice has received considerable attention in the field of organizational behavior because it generates a variety of negative outcomes. Among possible negative consequences, customer-directed sabotage is the most common reaction, which impacts individuals’ well-being and the prosperity of organizations. To minimize such negative consequences, researchers have sought to identify boundary conditions that could potentially attenuate the occurrence of customer-directed sabotage. In this study, we explore potential attenuation effects of emotional stability and attentiveness on the customer injustice–sabotage linkage. The results showed emotional stability and attentiveness moderate the relationship between customer injustice and customer-directed sabotage. Specifically, the representatives with higher (vs. lower) emotional stability or higher (vs. lower) attentiveness are less likely to engage in customer-directed sabotage when they experience customer injustice. Moreover, there is a three-way interaction among daily customer injustice, emotional stability, and attentiveness that predicts daily customer-directed sabotage. Theoretical and practical contributions, limitations, and directions for future development are also discussed.

## Introduction

*Customer injustice* is defined as poor-quality, unfair treatment that employees experience during service interactions received from customers, and examples of customer injustice include verbal aggression (e.g., yelling), disrespect (e.g., interrupting mid-sentence), and ignorance (e.g., refused to listen; Skarlicki et al., 2008; Wang et al., 2011; Shao and Skarlicki, 2014). In the last decade, customer injustice has been a primary research interest among organizational justice scholars because it generates a variety of negative outcomes. Examples of such negative consequences include emotional exhaustion (Grandey et al., 2007), emotional labor (Rupp and Spencer, 2006), turnover intention (Holmvall and Sidhu, 2007), and customer-directed sabotage (Skarlicki et al., 2008, 2016; Wang et al., 2011; Song et al., 2021). Among these possible negative consequences, customer-directed sabotage which is defined as employees’ counterproductive work behaviors which intentionally harm customers’ interests (Wang et al., 2011) has received considerable attention for two main reasons. First, the relationship is well supported by a strong theoretical foundation—namely, the target similarity effect. The main tenet of this theory is that the source of the injustice is likely to become a target of retaliatory behavior (Lavelle et al., 2007). Based on this theoretical background, the customer injustice literature has consistently asserted that customer-directed sabotage is the most common reaction among frontline employees mistreated by customers (e.g., Skarlicki et al., 2008, 2016; Wang et al., 2011). Second, the customer-directed sabotage literature has found that employees’ sabotage behavior generates a series of negative accompanying consequences that impact individuals’ well-being and the prosperity of organizations. That is, previous research has found that employee sabotage negatively affects frontline employees’ individual performance and overall well-being (Skarlicki et al., 2008; Wang et al., 2013). Furthermore, it damages the organization’s relationship with customers, harming customer loyalty and causing negative word of mouth, which may ultimately damage the organization’s profitability and future growth (Anderson et al., 1997; Harris and Ogbonna, 2002). To find ways to minimize such negative consequences, organizational justice scholars have sought to identify boundary conditions that could potentially attenuate the occurrence of customer-directed sabotage. In the next section, we review a variety of boundary conditions which have been explored in customer injustice research, including moral identity, cultural value differences, supervisory fairness, and emotion−/resource-based moderators.

## Literature Review

As described above, a growing body of research on the link between customer injustice and workplace sabotage has extensively tested the moderation effects of individual characteristics and situational factors. Skarlicki et al. (2008), for example, found that two components of moral identity—namely, symbolization and internalization—attenuate call center representatives’ workplace sabotage even if the individuals have been unjustly treated by customers. That is, individuals who have high symbolization are more likely to explicitly manifest their moral concerns by responding brusquely (i.e., workplace sabotage) when they are mistreated by customers. On the other hand, people with low internalization have a tendency not to punish transgressors in the event of customer injustice. In light of such findings, the authors hypothesized that the positive association between customer injustice and customer-directed sabotage would be most evident among those employees who have both high symbolization and low internalization.

Furthermore, other line of research has shown that both emotion- and resource-based moderators could attenuate the relationship between customer injustice and customer-directed sabotage (Wang et al., 2011). From the emotion-based perspective, a high level of negative affectivity exacerbates the link between customer injustice and sabotage; however, employees who have high self-efficacy in emotional regulation are less likely to engage in workplace sabotage even when they perceive injustice from customers. From the resource-based perspective, the authors further suggested that three types of resource-based moderators—namely, job tenure, service rule commitment, and supervisory support—may influence the relationship between customer injustice and workplace sabotage. They argued that frontline employees who have more tenure, higher commitment, and better/more supervisory support were less likely to conduct sabotage in response to customer injustice because they can draw supplementary resources from those resource reservoirs.

Comparing North American and East Asian hotel industry employees, research found that cultural value differences in two geographical locations (i.e., Canada and China) affect frontline employees’ behavioral pattern of customer-directed sabotage (Shao and Skarlicki, 2014). In particular, the employees with high (vs. low) individualism are more likely to engage in customer-directed sabotage when they are mistreated by customers. The authors explained the finding based on cross-cultural theory (Hofstede, 2001), claiming that cultural background affects individuals’ identity formulation (e.g., individualistic identity), ultimately influencing frontline employees’ choice of behavior in the event of customer mistreatment. That is, people who have lived within an individualistic culture are more likely to develop a self-focused attitude oriented toward high concern for self. Therefore, they are likely to demonstrate more active and direct reactions under stressful circumstances, which ultimately leads them to engage in more customer-directed sabotage.

A multifoci justice perspective proposes that more than one source of justice resource can simultaneously affect an individual’s justice perception and behavior (Lavelle et al., 2007). Based on such a theoretical foundation, recent customer injustice research has found that a low level of supervisory fairness strengthens/enhances the positive association between customer injustice and customer-directed sabotage, arguing that such a strengthening effect can be worsened due to multiple sources of unfairness (i.e., customer injustice and low supervisory fairness) concurrently affecting employees’ workplace behavior (Skarlicki et al., 2016).

Although previous research on customer injustice–sabotage linkage has explored a variety of moderation effects as described above, this study will test additional moderation effects worthy of further exploration for the following reasons. First, relatively little attention has been given to how personality traits and emotional state can affect customer-directed sabotage, especially *via* a compounding moderation effect of personality and emotion (for exceptions, see Skarlicki et al., 1999; Barclay et al., 2005). This gap in the research is notable because previous research has shown that individuals’ emotional state and personality traits are closely related to each other and that such an association can provide a syntagmatic influence on people’s subsequent workplace behaviors (Izard et al., 1993; George, 2000; Rubin et al., 2005). Therefore, the first goal of this study is to explore (a) whether a certain personality trait (i.e., emotional stability) and emotional state (i.e., attentiveness) individually attenuate the causal relationship and (b) a three-way interaction combining the moderation effect of emotional stability and attentiveness to determine the existence of a stronger moderation effect.

Second, this study urges scholars to pay more attention to the role of positive emotion while exploring the relationship between customer injustice and customer-directed sabotage. When customer injustice studies have examined the impact of an individual’s emotional state on his or her aggressive behavior, the emphasis has been on exploring how negative emotion and affectivity impact employees’ subversive workplace behavior (e.g., Skarlicki et al., 1999; Fox et al., 2001; Penney and Spector, 2005; Wang et al., 2011; Walker et al., 2014). Such an exclusive focus on negative emotion and its impact on workplace aggression create a research gap concerning the role of positive emotion in generating favorable workplace outcomes and reducing aggressive behavior (Staw et al., 1994; Twenge et al., 2007). Therefore, the second goal of this study is to explicate how a positive emotion, specifically attentiveness, can moderate the positive association between customer injustice and customer-directed sabotage. Attentiveness was chosen as an emotion-based moderator in this research because previous research has shown a strong connection between attentiveness and workplace behavior. That is, individuals with a high level of attentiveness tend to be more concerned about their job and performance; they therefore tend to exhibit more prosocial behavior and less antisocial behavior (Lee and Allen, 2002; Rodell and Judge, 2009). Therefore, this research expects that individuals with high attentiveness will be less likely to commit customer-directed sabotage, as it would jeopardize their job and future performance.

From a methodological standpoint, this study uses daily measurements to effectively capture short-term interactions between customers and employees. Using daily measurement to explore the moderation effects is important for the following reasons. First, an individual’s state of emotion, workplace experience, and behavioral patterns vary from moment to moment (Ohly et al., 2010). Therefore, it is essential to record momentary within-person changes over time, which can most effectively be accomplished through daily observation (Bolger et al., 2003). Second, research has shown that the human brain has limited capacity to remember. Therefore, a long-term survey design (e.g., at monthly intervals) cannot effectively capture the short-term dynamics of interpersonal interactions due to such limited memory capacity (Zapf et al., 1996). Based on such rationales, this study suggests a daily-interval, repeated measurement is best suited to explore customer–employee interactions in the workplace. From a managerial perspective, the findings in this study can be applied as a guideline for recruitment processes in the service industry to select the employees who are least likely to commit customer-directed sabotage due to effective regulation of their emotions in the workplace.

## Theory and Hypothesis Development

In the present study, we utilize affective event theory (AET, Weiss and Cropanzano, 1996) as a theoretical framework to explain the linkage between customer injustice and customer-directed sabotage. Besides AET, multiple other theories have been presented to explicate the customer injustice–sabotage linkage, including the moral perspective of justice (Folger, 2001), the norm of reciprocity (Gouldner, 1960), and conservation of resources theory (Hobfoll, 1989, 2001). Among the available theoretical backgrounds, this study suggests the AET as the most appropriate explanatory mechanism because (a) AET’s emotion-based explanatory mechanism provides a good reason for introducing emotional state as a moderator, and (b) it provides a theoretical basis for using a daily study design.

The main tenet of AET is that a typical event in the workplace may generate specific emotions in an employee (Weiss and Cropanzano, 1996), ultimately underlying such individual affect-driven attitudinal, emotional, or behavioral outcomes as job dissatisfaction (Wegge et al., 2006), increased emotional labor (Rupp et al., 2008), counterproductive work behavior (Matta et al., 2014), and employee incivility (Walker et al., 2014). AET studies have consistently found that individuals’ emotional state can influence their affect-driven, reactive workplace behavior within a short timeframe. For example, research has shown that individuals’ emotional state can fluctuate rapidly over time, and the unstable nature of emotion can influence individuals’ attitudinal and behavioral outcomes in the workplace (Weiss and Cropanzano, 1996).

In addition, previous research has found that “organizational members’ cognition and behavior at work are much more likely to be affected by the way they feel on a moment-to-moment basis than by stable belief systems or previously formed attitudes about those workplace events” (Ashton-James and Ashkanasy, 2008, p. 9). Thus, the short-term oriented, time-dependent nature of AET could effectively explicate the momentary emotional and behavioral changes of employees in the workplace. This constitutes our rationale for why AET is the ideal theoretical lens for our daily-interval study of the link between customer injustice and customer-directed sabotage.

### Customer Injustice and Customer-Directed Sabotage

Organizational justice scholars have often used AET as a theory to explicate the relationship between workplace aggression and employees’ corresponding negative outcomes (e.g., Rupp and Spencer, 2006; Matta et al., 2014; Walker et al., 2014). For example, a specific type of workplace event, such as perceiving unfairness, instigates frontline employees’ negative emotion, which in turn generates subsequent emotional outcomes (e.g., emotional labor) when the employees must comply with the organization’s displayed rules (Rupp and Spencer, 2006). As another example, customer incivility leads to employees’ injustice perception and negative emotion, which in turn makes the employees desire to retaliate against the harm-doing transgressor, thereby triggering employee incivility toward the source of the original incivility (Walker et al., 2014).

In the present study, it is postulated that the relationship between frontline employees’ daily experience of customer injustice and daily customer-directed sabotage can also be explained by AET. Previous call center research has found that frontline employees experience, on average, 10 instances of customer mistreatment per day (Grandey et al., 2004). Such excessive amounts of unfair treatment could generate negative emotions (e.g., anger and frustration), ultimately affecting employees’ behavioral outcomes. For example, research has shown that frontline employees’ feelings of anger resulting from customer mistreatment are the main cause of retaliatory actions (Barclay et al., 2005). We therefore theorized that frontline employees’ response to unfair treatment from customers will generate feelings of anger, which eventually produces the customer-directed sabotage.

*Hypothesis 1*: Daily customer injustice is positively associated with daily customer-directed sabotage.

### The Moderating Effect of Emotional Stability

*Emotional stability* is defined as the individual personality trait of being stable, self-poised, and independent (Ehrhart et al., 2008). According to personality trait research, individuals with high emotional stability tend to effectively manage their life events due to high ability to control their emotions and moods (Newsome et al., 2000). Emotional stability is one of the Big Five personality traits, which include extraversion, agreeableness, conscientiousness, openness to experience, and emotional stability (Goldberg, 1999). In the present paper, we focus on emotional stability as a moderator because the positive aspects of emotional stability can attenuate the occurrence of customer-directed sabotage. Traditionally, personality trait research often focused on the impact of *neuroticism*, another way to describe a low level of emotional stability, and its impact on people’s behavioral outcomes. Neuroticism often refers to the likelihood of experiencing negative affect in the workplace (McCrae, 1990). That is, people with high levels of neuroticism are more likely to experience fear, anger, anxiety, and hostility (McCrae and Costa, 1986; Gunthert et al., 1999). Research has consistently shown neuroticism to be the most common construct that aggravates individuals’ aggressive behavior (Caprara et al., 1996; Bettencourt et al., 2006; Flaherty and Moss, 2007; Egan and Lewis, 2011).

Instead of focusing on neuroticism, however, this study focuses on the positive side—how frontline employees’ high level of emotional stability can weaken the positive relationship between customer injustice and customer-directed sabotage. That is, when individuals with high emotional stability perceive unfairness, they are less likely to express inappropriate emotion and negative attitude toward the source of injustice than individuals who have low emotional stability (Milam et al., 2009). In other words, higher emotional stability enables individuals to effectively control their own emotions and thus be more likely to handle the provoking incident in an objective and cool-headed way. Therefore, call center representatives with high levels of emotional stability are less likely to conduct sabotage because their regulation of emotion prevents them from responding to an inflammatory event (i.e., customer injustice) in an inappropriate way.

*Hypothesis 2*: Emotional stability moderates the positive relationship between daily customer injustice and daily customer-directed sabotage; the relationship is less (vs. more) pronounced for employees who have high (vs. low) levels of emotional stability.

### The Moderating Effect of Attentiveness

*Attentiveness* is defined as an individual’s feeling of alertness, concentration, and determination in relation to jobs and duties (Rodell and Judge, 2009). Attentiveness, an example of a positive emotional state, is classified as one of the basic low-order forms of positive affectivity, along with joviality and self-assurance (Watson and Clark, 1994). Attentiveness is also well known for its strong relationship with job engagement (Watson and Tellegen, 1985). Organizational engagement research has found that individuals with high work engagement are more likely to conduct better customer service and less likely to display counterproductive work behavior when faced with workplace mistreatment (Harter et al., 2006; Fine et al., 2010). Furthermore, if a workplace has many employees with high levels of work engagement, employees may be infected by their colleagues’ enthusiasm for their work and duties and consequently can conduct more organizational citizenship behavior and less counterproductive work behavior (Bakker, 2008).

Based on these findings, this study predicts that call center representatives who have high levels of attentiveness are less likely to conduct customer-directed sabotage even if they perceive unjust treatment from customers. Frontline employees with higher attentiveness will be more enthusiastically involved in their job-related tasks and goals, and such strong engagement in one’s duties makes them less likely to exhibit counterproductive work behavior including sabotage. A main duty of service sector workers is to serve and interact with customers to maximize their performance and productivity. Therefore, we theorize that call center representatives who possess higher work engagement are less likely to commit workplace sabotage during interaction with customers because they care greatly about their work. As a result, they are less likely to direct harmful behavior toward customers who are the resource of their daily performance.

*Hypothesis 3*: Attentiveness moderates the positive relationship between daily customer injustice and daily customer-directed sabotage; this relationship is less (vs. more) pronounced for employees who have high (vs. low) levels of attentiveness.

### Three-Way Interaction Between Emotional Stability and Attentiveness

Previous studies on the relationship between personality and emotion have posited a strong connection between individual emotional states and personality traits (Izard and Malatesta, 1987; Malatesta, 1990; Smith and Lazarus, 1990; Watson and Clark, 1992; Izard et al., 1993). For example, extraversion has a strong correlation with positive affect, while low emotional stability has a strong correlation with negative affectivity (Costa and McCrae, 1992). There are two streams of research exploring the causal relationship between personality traits and emotional state. On the one hand, it has been argued that individuals’ patterns of emotional response contribute to their formulation of emotion-based personality traits (Izard et al., 1993). From this perspective, *personality trait* is defined as a coherent patterning of emotions over a certain period of time, and an accumulation of such consistent behavioral patterns that ultimately displays the specific personality characteristics (Revelle and Scherer, 2009). By contrast, the other stream of personality trait research has found that individuals’ personality characteristics influence their emotional status, which ultimately affects people’s behavior. Specifically, individuals’ unique personality traits, including intuitive knowledge about the self, can affect momentary emotional responses (Smith and Lazarus, 1990). In light of such strong causal ties between personality traits and momentary emotional state or vice versa, it is reasonable to expect two boundary conditions, emotional stability and attentiveness, can jointly moderate the linkage between customer injustice and customer-direct sabotage.

As described earlier, employees with high levels of emotional stability will have a strong ability to control their emotion and are thus able to suppress their desire to commit customer-directed sabotage although they have been mistreated by customers. Furthermore, the employees with higher attentiveness will be more likely to enthusiastically engage in their job-related tasks and performance. Therefore, they would prevent the occurrence of customer-directed sabotage because customers are the major resources of their performance.

In the present study, we theorize that there is a combined moderation effect between emotional stability and attentiveness by a three-way interaction formed in the relationship between customer injustice and customer-directed sabotage. Previous research has shown that individual differences in personality traits can directly influence the creation of emotional states (Smith and Lazarus, 1990). To support such a connection between personality trait and emotional state, Tellegen (1985) postulated that individuals with high levels of positive emotionality are more likely to have high levels of attentiveness. That is, high emotional stability enabled individuals to pay more attention to job-related issues, which implies that they become more attentive to their job and performance. Tellegen’s argument suggests that frontline employees with high emotional stability are more likely to generate a more positive emotional state—namely, attentiveness. Based on such aspects of emotional stability and attentiveness, we expect that customer-directed sabotage will be least pronounced among employees who have both high emotional stability and high attentiveness. That is, higher emotional stability helps them to better control their negative emotions arising from customer injustice, thereby effectively suppressing their inclination to commit customer-directed sabotage. At the same time, frontline employees with higher emotional stability can generate a more attentive attitude toward their job and performance, and they are therefore less likely to engage in customer-directed sabotage because such behavior can impair their future performance.

*Hypothesis 4*: The relationship between daily customer injustice and daily customer-directed sabotage is least pronounced for employees who simultaneously have high (vs. low) levels of both emotional stability and attentiveness.

## Methodology

### Data Collection Process

A questionnaire for this study was generated in English first and then translated into Korean using the translation–back translation technique (Brislin, 1990). Paper-and-pencil-based surveys were administrated at 10 insurance call centers located in South Korea. Participants were encouraged to finish all 10 daily surveys upon completion of their shift each day at 6 pm. On the first day of the surveys, participants were asked to answer some basic questions, including demographic information such as age, education, tenure, and annual income for use as control variables. From the second to the tenth day, the two daily-based variables—customer injustice and customer-directed sabotage—were measured at multiple time points using the same questionnaire. Then, 50 items of International Personality Item Pool–Five Factor Model (IPIP-FFM; Goldberg, 1999) were administered to participants to measure their personality traits. As a result, 2,140 level 1 samples were obtained out of total possible 2,331 level-1 samples (259 × 9 days), after omitting cases with missing values on any variables.

### Participants and Demographics

Four hundred call center telemarketing representatives from 10 different insurance companies were invited to participate by taking a total of 10 surveys daily for 10 days; 309 participants returned the questionnaires, giving a response rate of 83%. Some of the daily surveys did not provide the identifiable information in each survey and 50 of the completed daily surveys could not be connected; therefore, the final sample for this study was 259 participants. The analysis of the 259 participants indicated that the average age was 39.00 years old (*SD* = 9.09), and 83% were female, with an average of 1.16 years of tenure (*SD* = 1.55) at their current organization.

### Measures

All measures that were used in this study were validated and selected from previously published peer-reviewed journals. Some measures were slightly modified for the daily survey format by adding additional instructions or explanations, such as “please recall your experience during today’s working hours.”

#### Daily Customer Injustice

Skarlicki et al.’s (2008) validated 8-item scale was used to evaluate employees’ daily perception of changes in customer injustice (to find complete items, please see the Appendix). The measure was assessed by using a 5-point Likert-type response scale ranging from 1 *(never)* to 5 *(frequently)*. After finishing their work (at 6:00 p.m.), respondents were asked to check the frequency of their experience of customer injustice during their working hours. Sample questions from this measure include, “During work hours today, a customer spoke aggressively to you.” These 8 items were averaged to create the index of customer interpersonal injustice (*α* = 0.85).

#### Daily Customer-Directed Sabotage

Call center representatives’ daily sabotage was assessed with Skarlicki et al.’s (2008) 5 validated items, which use a 5-point Likert-type response scale ranging from 1 *(never)* to 5 *(frequently, more than 7 times per day)*. Sample questions of this measure include, “During work hours today, I purposely disconnected the call” (*α* = 0.86), and all items are listed in the Appendix.

#### Emotional Stability

The Big Five personality traits were measured with the IPIP FFM (Goldberg, 1999). A total of 50 questions were asked to measure the five dimensions of employee personality traits (i.e., extraversion, agreeableness, conscientiousness, emotional stability, openness to experience) using a 7-point Likert-type response scale ranging from 1 *(strongly disagree)* to 7 *(strongly agree)*. Emotional stability, one of the dimensions of the Big Five personality traits, consisted of 10 questions, and sample questions for emotional stability include, “I have frequent mood swings” *(reversed code)* and “Take time for others” (*α* = 0.90).

#### Attentiveness

Attentiveness was measured based on the PANAS-X scales (Watson and Clark, 1994). Participants were instructed to read the given words (i.e., alert, attentive, concentrating, determined) and indicate to what extent they felt that way over the past few weeks. The frequency of feeling on the given four words were measured by using a 7-point Likert-type response scale from 1 *(Never)* and 7 *(Always)*. The items were averaged to generate the index of attentiveness (*α* = 0.73).

#### Control Variables

Participants’ age, education level, tenure, and annual income were controlled for the following reasons. Age was controlled because it is correlated with workplace aggression (Glomb and Liao, 2003). According to Douglas and Martinko (2001), level of education is positively associated with individuals’ counterproductive work behavior in the workplace. Tenure was also controlled because a previous study found that longevity of one’s current affiliation is negatively associated with workplace sabotage behavior (Sims, 2002). Lastly, to clearly investigate the moderating effects of attentiveness and emotional stability on customer injustice–sabotage linkage, the remaining four dimensions of the Big Five personality traits (i.e., conscientiousness, extraversion, agreeableness, and openness to experience) and two other positive emotional state (i.e., self-assurance and joviality) dimensions from the PANAS-X were also controlled.

## Analytical Strategy

The multi-level dataset for this study was analyzed in two steps: (1) preliminary analyses and (2) main analysis including lower-(daily) level main effect, cross-level two-way and three-way interactions using multilevel hierarchical regression analysis.

### Preliminary Analyses

As a first step, the means, standard deviations, and inter-correlations (i.e., Cronbach alpha values) among study variables were examined to evaluate the suitability of the proposed variables. The Cronbach alpha values for all variables used in this study were found to be between 0.73 and 0.90, which satisfied the criteria for a reasonably acceptable reliability (*α* = 0.70; Nunnally and Bernstein, 1994; Table 1).

**Table 1:** Means, Standard Deviations, Correlations, and Reliability Estimate for Study Variables

Variables	*M*	*SD*	1	2	3	4	5	6	7	8	9	10	11	12	13	14
1. Age (T1)	39.00	9.09	–													
2. Education^a^ (T1)	1.73	0.82	0.00	–												
3. Tenure (T1)	1.16	1.55	0.11^**^	−0.00	–											
4. Annual Income^b^ (T1)	1.06	0.99	0.13^**^	0.14^**^	0.15^**^	–										
5. Conscientiousness (T10)	4.71	0.76	0.26^**^	0.06^**^	0.03	0.09^**^	(0.80)									
6. Extraversion (T10)	3.93	0.75	−0.18^**^	0.05^*^	−0.02	−0.01	−0.07^**^	(0.80)								
7. Agreeableness (T10)	4.70	0.68	0.03	0.03	−0.07	−0.05^*^	0.22^**^	0.29^**^	(0.74)							
8. Openness to change (T10)	4.30	0.67	−0.06^**^	0.14^**^	−0.02	0.04	0.19^**^	0.38^**^	0.17*^*^	(0.78)						
9. Self-assurance (T10)	3.81	0.98	−0.13^**^	0.08^**^	0.06	0.07^**^	0.10^**^	0.27^**^	0.02	0.24^**^	(0.83)					
10. Joviality (T10)	4.16	0.96	−0.18^**^	0.14^**^	0.00	0.00	0.14^**^	0.25^**^	0.17^**^	0.28^**^	0.72^**^	(0.88)				
11. Emotional stability (T10)	4.10	0.98	0.13^**^	0.13^**^	−0.06	−0.04	0.26^**^	−0.01	−0.07^**^	0.03	0.23^**^	0.21^**^	(0.90)			
12. Attentiveness (T10)	3.96	0.88	0.01	0.09^**^	0.06	0.18^**^	0.19^**^	0.08^**^	0.15^**^	0.11^**^	0.59^**^	0.52^**^	−0.04^*^	(0.73)		
13. Daily CIJ^c^ (T2-9)	2.07	0.80	−0.08^**^	0.03	−0.00	−0.02	−0.06^**^	−0.11^**^	−0.10^**^	−0.11^**^	0.06^**^	0.04^*^	−0.06^**^	0.14^**^	(0.85)	
14. Daily SABO^d^ (T2-9)	1.18	0.46	−0.11^**^	−0.06^**^	0.10	0.06^**^	−0.19^**^	0.06^**^	−0.06^**^	−0.06^**^	0.07^**^	−0.03	−0.06^**^	−0.00	0.29^**^	(0.86)

a Education is coded as 0 (middle school or less), 1 (high school), 2 (some college or two-year college degree), and 3 (four-year bachelor’s degree or more).

b Annual income is coded as 0 *(less than CAD 20 K)*, 1 *(CAD 20-40 K)*, 2 *(CAD 40-60 K)*, 3 *(CAD 60-80 K)*, 4 *(CAD 80-100 K)*, 5 *(CAD 100-120 K)*, and 6 *(more than CAD 120 K)*; T1 = survey day1; ^c^CIJ means customer injustice; ^d^SABO means customer-directed sabotage; T2-9 = survey from day2 to day9; T10 = survey day10; *N* for Level 1 *(within-person level)* is 2140; *N* for Level 2 *(between-person level)* is 259; To calculate the between-person correlations, we averaged within-person level constructs’ (i.e., daily CIJ, daily and daily SABO) during eight daily surveys and then computed the between-person level correlations across individuals.

* *p* < 0.05;

** *p* < 0.01 (2-tailed, Likewise).

In addition, variables used in this study will be grand-mean centered for the upper (between-person) level and group-mean centered for the lower (within-person) level (Ohly et al., 2010). Furthermore, to confirm the rationale for performing multilevel modeling, the intra-class correlation (ICC_1_) was calculated. We found that the ICC_1_ value was 0.72, which implied that about 72% of the variance of daily customer-directed sabotage was explained by individual difference. An ICC_1_ value of 0.70 or higher can be considered as acceptable; therefore, it provided sufficient rationale to conduct multi-level analysis for this study (Klein et al., 2000).

Because previous research has found that there are relatively high correlations among the Big Five personality traits (Paunonen and Jackson, 2000), this study checked for multicollinearity among all variables used in this study using tolerance values and variance inflation factors (VIF). The primary analysis found that the VIF values of all variables used in this study are located between 1.1 and 2.7. Multicollinearity was ruled out since our values were all below the suggested cut-off VIF of approximately 5.3 (Hair et al., 1998).

As the last step of the preliminary analysis, confirmatory factor analysis (CFA) was conducted for each level (McDonald, 1993; Mathisen et al., 2006). That is, research showed that two separate CFAs are ideal (one for the within-person level and the other for the between-person level) to clearly assess the goodness of fit on two distinct levels. The confirmatory factor analysis (CFA) was conducted using Mplus 6.0 (Muthén and Muthén, 2012). The results showed that the two-factor measurement model provided a reasonably good fit for the data at both between-individual level and within-individual levels (within-person level: *χ*^2^ = 233.78, *p* < 0.001, CFI = 0.94, RMSEA = 0.08, SRMR = 0.06; between-person level: *χ*^2^ = 359.64, *p* < 0.001, CFI = 0.95, RMSEA = 0.08, SRMR = 0.06; Table 2).

**Table 2:** Fit Indices for alternative measurement models (Study 3).

Measurement models		*χ* ^2^	*df*	*χ*^2^/*df*	CFI	TLI	SRMR	RMSEA
1-factor model^a^	Between-person	805.61	20	40.28	0.87	0.82	0.09	0.13
2-factor model^b^	Between-person	359.64	19	18.93	0.95	0.93	0.06	0.08
1-factor model^c^	Within-person	1,359.81	14	97.13	0.62	0.44	0.16	0.16
2-factor model^d^	Within-person	233.78	13	17.98	0.94	0.90	0.06	0.08

*N* = 259. CFI = comparative fit index, TLI = Tucker-Lewis index, SRMR = standardized root mean square residual, RMSEA = root mean square error of estimators.

a Emotional stability and attentiveness loaded on a single factor.

b Emotional stability loaded on one factor, and attentiveness loaded on second factor.

c Daily customer interpersonal injustice and daily customer-directed sabotage loaded on a single factor.

d Daily customer interpersonal injustice loaded on one factor and daily customer-directed sabotage loaded on second factor.

### Main Analysis

To conduct the multi-level hierarchical regression analysis, main-effect and cross-level moderating interactions were tested using SAS 9.4 software (Bauer and Curran, 2005). The multilevel data was analyzed through a three-step process. First, the within-person variance between daily customer interpersonal injustice and daily customer-directed sabotage was analyzed to determine whether a within-person main effect exists between daily customer injustice and daily customer-directed sabotage. Second, it was tested whether between-person variables (i.e., emotional stability and attentiveness) individually have a cross-level moderation effect on the within-person daily main effect. Lastly, the existence of a three-way interaction among daily customer injustice, emotional stability, and attentiveness on the frontline employees’ daily customer-directed sabotage was tested.

## Results

Hypothesis 1 stated that a positive relationship exists between daily customer injustice and daily customer-directed sabotage. Model 1 in Table 3 shows that the effect of customer injustice on customer-directed sabotage was positive and significant (γ_10_ = 0.12, *p* < 0.001). Furthermore, the results show that the variance of the random slope was also statistically significant (τ_11_ = 0.04, *p* < 0.001). This finding implies that employees who perceive injustice from customers are more likely to engage in sabotage behavior toward customers on that particular day. Table 3 presents all values of within-person level parameter estimates, including fixed intercept and random slope values, to predict customer-directed sabotage.

**Table 3:** Joint effects of daily customer interpersonal injustice, emotional stability, and attentiveness on daily customer-directed sabotage.

Level and variables	Null model	Model 1 (H1)	Model 2 (H2)	Model3 (H3)	Model 4	Model 5 (H4)
Level 1						
Intercept (γ_00’_)	2.05^***^	1.90^***^	1.98^***^	1.91^***^	1.98^***^	1.99^***^
Daily Customer Injustice (CIJ; γ_10’_)		0.12^***^	0.11^***^	0.12^***^	0.12^***^	0.12^***^
Level 2						
Emotional Stability (γ_01’_)			0.01		0.02	0.02
Attentiveness (γ_02’_)				−0.01	−0.02	−0.02
Cross-level Interactions						
Daily CIJ × Emotional Stability (γ_11’_)			−0.04^*^		−0.03	−0.01
Daily CIJ × Attentiveness (γ_21’_)				−0.05^*^	−0.04^*^	−0.04^*^
Emotional Stability × Attentiveness (γ_21’_)					−0.04^*^	−0.03
Daily CIJ × Emotional Stability × Attentiveness (γ_22’_)						−0.03^*^
Variance components						
Within-subject (Level 1) variance (σ^2^)	0.06^***^	0.05^***^	0.05^***^	0.05^***^	0.05^***^	0.05^***^
Intercept (Level 2) variance (τ_00_)	0.16^***^	0.10^***^	0.11^***^	0.11^***^	0.11^***^	0.10^***^
Slope (Level 2) variance (τ_11_)		0.04^***^	0.04^***^	0.04^***^	0.04^***^	0.04^***^
ICC	0.72					

N_time_ for Level 1 (within-person level) is 2,140; N for Level 2 (between-person level) is 259.

* *p* < 0.05;

*** *p* < 0.001 (2-tailed).

**Figure 1:**
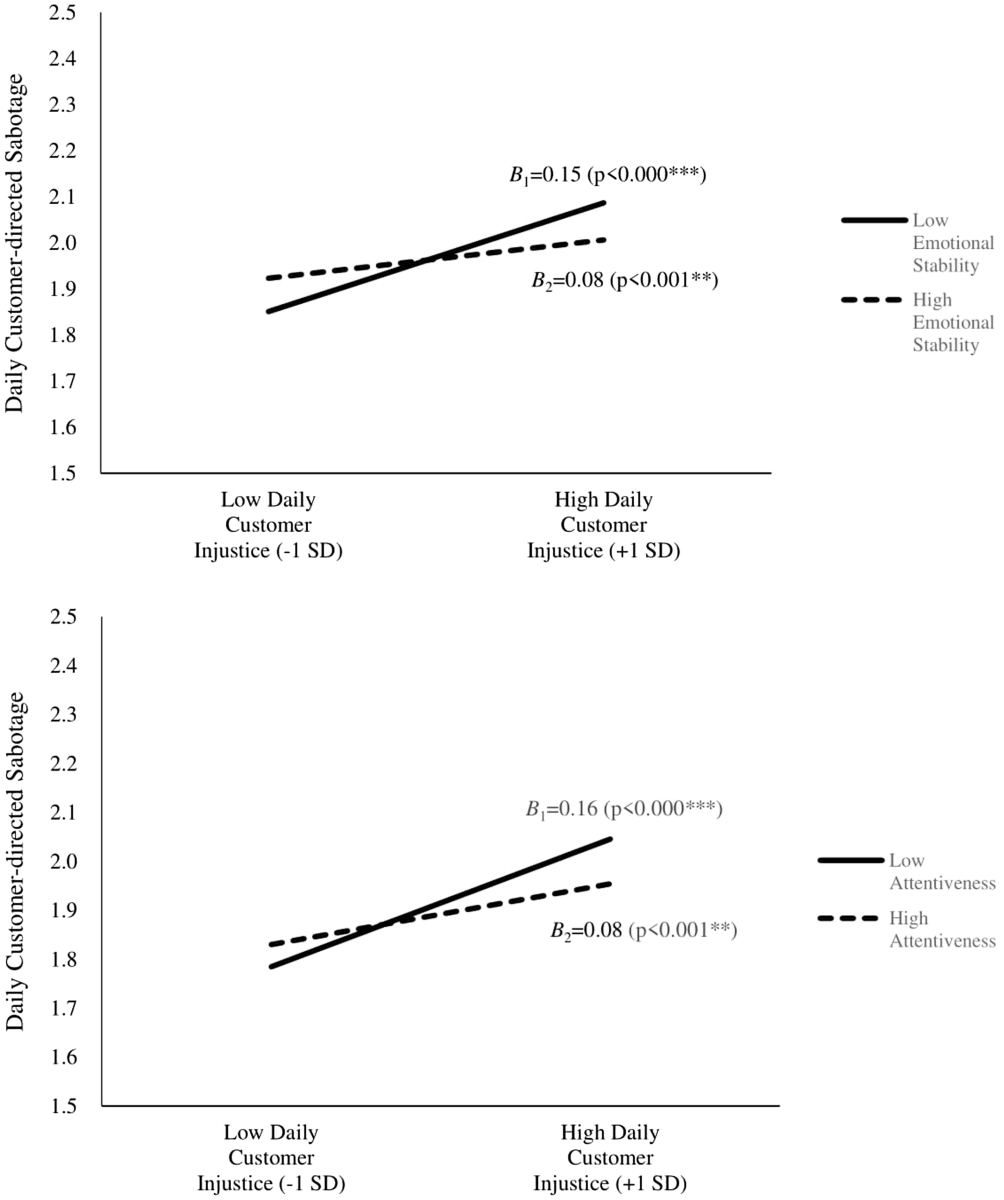
Two-way interactions.

Hypothesis 2 proposed that there would be a cross-level moderation effect of emotional stability, which moderates the within-person level main effect between daily customer injustice and daily customer-directed sabotage. The moderating effect of emotional stability was found to be negative and significant (γ_11_ = −0.04, *p* < 0.05), thus supporting Hypothesis 2. Similarly, our next hypothesis was that attentiveness moderates the relationship between daily customer injustice and daily customer-directed sabotage. This moderation effect was also found to be negative and significant (γ_21_ = −0.05, *p* < 0.05); therefore, Hypothesis 3 was also supported. These two hypotheses reveal that call center representatives with either high emotional stability or high attentiveness tend to commit less daily customer-directed sabotage under higher daily customer mistreatment conditions. Figure 1 presents cross-level moderation effects of emotional stability and attentiveness.

**Figure 2:**
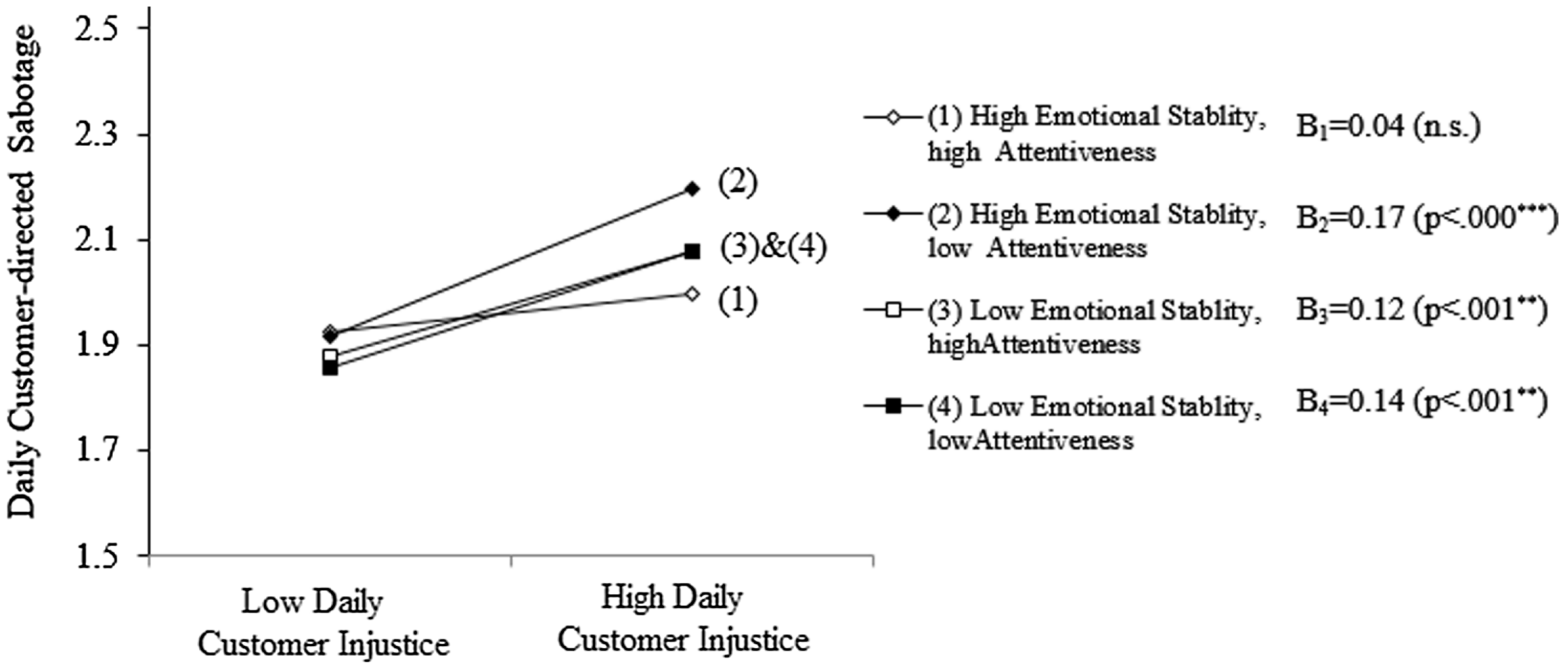
Three-way interactions.

Finally, Hypothesis 4 predicted that a three-way interaction among daily customer injustice, attentiveness, and emotional stability would predict frontline employees’ daily customer-directed sabotage. Model 5 in Table 3 shows that the three-way interaction effect was significant (γ_22_ = −0.03, *p* < 0.05), predicting daily employee sabotage. Simple slope analyses demonstrated that the call center representatives with high levels of attentiveness and emotional stability were least likely to conduct customer-directed sabotage (*B*_1_ = 0.04, *p* < 0.18 n.s.) among four possible cases. Figure 2 shows the three-way interaction among the aforementioned variables.

## Discussion

The recent burgeoning of research on customer injustice–sabotage linkage has looked extensively for boundary conditions to minimize the ill-effect of customer injustice. Such boundary conditions include individual differences (e.g., moral identity, emotional intelligence, and self-efficacy), situational factors in the workplace (e.g., supervisory fairness and perceived organizational support), or geographical location in different cultures (Skarlicki et al., 2008, 2016; Wang et al., 2011; Lee and Ok, 2014; Shao and Skarlicki, 2014). In the present study, we additionally introduced two boundary conditions, emotional stability and attentiveness, to explore their potential attenuation effects on the customer injustice–sabotage linkage. we believe that the inclusion of such new boundary conditions provides meaningful theoretical, methodological, and managerial implications.

First, from the theoretical standpoint, this study broadens the spectrum of possible boundary conditions to lessen the ill-effect of customer injustice. Although there have been many studies that explored the boundary conditions as listed above, we know very little about whether people’s emotions and personality traits can interact with each other, and how they can simultaneously affect the emergence and development of customer-directed sabotage among employees. Considering emotional state as one of the key predictors of people’s workplace behavior and the strong causal connection between emotion and personality traits, this study adds value to the sabotage research by expanding the scope of boundary conditions that influence frontline employees’ retaliatory behavior in the workplace. Furthermore, this study introduces AET as a theoretical framework that provides a good fit to the proposed moderation effects of emotion and personality traits. That is, AET focuses on how individuals’ emotional changes impact their workplace behavior, which provides a strong theoretical connection with the suggested emotion- and personality-based moderators in the present study.

Second, this study emphasizes the role of positive emotional state and its positive attenuation effect on customer-directed sabotage. In previous workplace aggression research, negative aspects of emotion and personality traits have received more attention as potential moderators than positive ones. For example, negative affectivity has been identified as a moderator that increases individuals’ workplace aggression (e.g., Penney and Spector, 2005; Wang et al., 2011; Walker et al., 2014). Similarly, neuroticism has always been one of the focal personality traits that predicts forms of workplace aggression, such as workplace incivility (Milam et al., 2009), counterproductive work behavior (Bowling et al., 2011), and employee service sabotage (Chi et al., 2013). In the present study, however, the focus is given to positive emotional states and personality traits—namely, attentiveness and emotional stability—to highlight their attenuating effects on customer-directed sabotage. This study explicitly addressed the positive personality trait and emotional state that can help employees to better cope with customer injustice and minimize the occurrence of customer-directed sabotage, providing theoretical and empirical details necessary to understand the role of personality traits and emotional state in ameliorating the ill effects of customer injustice.

From a methodological perspective, most studies on the relationship between customer injustice and customer-directed sabotage have utilized cross-sectional data (for an exception, see Wang et al., 2011, 2013), but such a research design cannot effectively capture the short-term dynamic nature of frontline employees’ emotional and behavioral changes and their relation to the customer injustice–sabotage linkage. This study increases the reliability of findings by introducing a daily study design to more precisely capture employees’ daily-interval emotional and behavioral changes. The advantages of such a design include minimization of retrospective bias, which occurs when a less fine-grained survey design is applied (Reis and Gable, 2000; Ohly et al., 2010). That is, fluctuations in individuals’ emotional and behavioral patterns throughout their daily routines cannot be accurately measured by either cross-sectional or longitudinal research designs due to limitations of human memory; therefore, neither design can precisely capture employees’ within-subject variation. Implementing a daily-interval study to explore the customer injustice and customer-directed sabotage relationship thus represents a considerable advancement in the rigorousness of empirical findings.

Furthermore, this study has numerous control variables, including general demographic information (age, education level, tenure, and annual income), the remaining Big Five personality traits (conscientiousness, extraversion, agreeableness, and openness to change), and other positive emotional states (joviality and self-assurance) to make the findings more parsimonious and rigorous. Such numerous control variables may counterintuitively imply that there are many potential opportunities to develop new emotion- and personality-combined moderation effects by introducing the remaining Big Five personality traits and positive emotional state constructs as explanatory variables, indicating a potential future research direction.

From a managerial perspective, this study provides an insight into how to manage the recruitment and selection process to improve customer service quality and minimize potential customer-directed sabotage. In a service industry, it is conceivable that frontline employees are repeatedly exposed to multiple instances of customer mistreatment throughout their working hours. Therefore, there is always the potential for occurrence of customer-directed sabotage. We suggest that organizations in the service industry should assess job candidates’ levels of attentiveness and emotional stability as part of the hiring process to predict the candidates’ ability to refrain from engaging in customer-directed sabotage.

### Limitations and Future Research Directions

Although this study makes significant contributions as discussed above, this study also has some limitations that must be outlined to benefit future research. First, the self-reported nature of the data raises the issue of common method variance (Podsakoff et al., 2003). However, individuals’ personality traits can hardly be measured by a third party, so this study must rely on self-reporting despite the recognized disadvantages. Therefore, future research should find alternative measures to minimize such concerns. For example, employees’ daily service sabotage could be observed by managers or coworkers, or observed *via* recorded conversations (with customers) during working hours.

Second, there may be an issue of generalizability, because the data was collected from South Korea, a country with a strongly collectivistic culture. Considering cultural differences moderate frontline employees’ willingness to retaliate in response to unjust treatment from customers (Shao and Skarlicki, 2014), another dataset from a geographic location with a more individualistic cultural climate (e.g., Canada) is recommended to lessen the concern of generalizability.

Moreover, given that our study was conducted in a Korean call center where a majority of call center representatives are female, we encourage future research that empirically evaluates the gender difference in other settings to get more in-depth knowledge of how and why gender difference could affect customer-directed sabotage and its boundary conditions.

In terms of future research direction, this study should additionally explore the mediation effect, especially the suggested theoretical foundation of AET. Considering that the main tenet of AET contains an emotion-based mediation process in its structure (i.e., an event generates discrete emotions within individuals, which in turn influence their behavioral patterns), future studies should examine this process. That is, applying AET makes more sense if discrete emotions (e.g., anger or frustration) mediate the main effect between customer injustice and customer-directed sabotage. Future research, therefore, should aim to collect additional data to test the suggested mediation effect and make a strong connection to the given theory.

In addition, this study only tested two specific emotion- and personality-based moderators, emotional stability and attentiveness, and this limited scope should be expanded in future studies. Future research should aim to explore how the remaining four types of personality traits (e.g., agreeableness, conscientiousness, openness to change, or extraversion) and additional low-level discrete emotions (e.g., self-assurance or joviality) may moderate the relationship between customer injustice and customer-directed sabotage. For example, negative discrete emotions such as fear, guilt, and sadness are strongly related to the development of the personality trait of neuroticism (Watson and Clark, 1992). Some researchers, furthermore, postulated a sequential linkage among emotion, cognition, and action that explicates people’s behavior and helps explain how individuals’ personality traits develop and evolve (e.g., Izard et al., 1993). Therefore, future studies should examine the causal relationship between discrete emotions and personality traits to better understand why and how emotional state and personality traits moderate the customer injustice and sabotage linkage.

To further improve insights into the daily customer injustice–sabotage relationship, future studies should aim to examine how the previous day’s (i.e., t − 1 day) customer injustice could influence employees’ next-day (i.e., t + 1 day) customer-directed sabotage by incorporating the literature on the spill-over effect, considering that mood in a certain domain could possibly transfer to another domain (Edwards and Rothbard, 2000). In addition, previous research has shown that individuals display different levels of rumination tendencies even when they have experienced similar levels of customer injustice (Wang et al., 2013). Therefore, future research should include how such individuals’ different levels of rumination might influence the relationship between customer injustice and customer-directed sabotage.

Although it is conceivable that customer injustice can mostly be directed to harm-doing customers based on target similarity effect, it will be also worthwhile to explore employees’ possibility of committing another form of retaliatory action toward different targets, including competitors, co-workers, and subordinates in the workplace. Future studies should aim to explore such possibilities by understanding its nature (for a review, see Chowdhury and Gürtler, 2015) to apply a different theoretical perspective (e.g., displaced aggression).

## Data Availability Statement

The original contributions presented in the study are included in the article/supplementary material, further inquiries can be directed to the corresponding author.

## Ethics Statement

The studies involving human participants were reviewed and approved by McGill University. The patients/participants provided their written informed consent to participate in this study.

## Author Contributions

This research was originally conducted as a part of YHS’s doctoral dissertation at McGill University, Desautels Faculty of Management. YHS wrote the original manuscript including conception, design, and statistical analysis, and JP revised the manuscript and reviewed the statistical analysis for the submission. Both authors read and approved the submitted version of manuscript.

## Conflict of Interest

The authors declare that the research was conducted in the absence of any commercial or financial relationships that could be construed as a potential conflict of interest.

## Publisher’s Note

All claims expressed in this article are solely those of the authors and do not necessarily represent those of their affiliated organizations, or those of the publisher, the editors and the reviewers. Any product that may be evaluated in this article, or claim that may be made by its manufacturer, is not guaranteed or endorsed by the publisher.

## Appendix

Customer Injustice Items (Skarlicki et al., 2008)

1. Refused to listen to you
2. Interrupted you: Cut you off mid-sentence
3. Made demands that you could not deliver
4. Raised irrelevant discussion
5. Doubted your ability
6. Yelled at you
7. Used condescending language (e.g., “you are an idiot”)
8. Spoke aggressively to you

Customer-directed Sabotage Items (Skarlicki et al., 2008)

1. Hung up on the customer.
2. Intentionally put the customer on hold for a long period of time.
3. Purposefully transferred the customer to the wrong department.
4. Purposefully disconnected the call.
5.Told the customer that you fixed something but did not fix it.
